# Geospatial analysis of dissolved nutrients dataset in the surface water of Karayar reservoir, Southern India

**DOI:** 10.1016/j.dib.2017.06.037

**Published:** 2017-06-24

**Authors:** N.S. Magesh, N. Chandrasekar, S. Krishnakumar

**Affiliations:** a Centre for Geotechnology, Manonmaniam Sundaranar University, Tirunelveli 627012, Tamil Nadu, India; b Department of Geology, University of Madras, Guindy Campus, Chennai 600025, Tamil Nadu, India

**Keywords:** Nutrients, Surface water, Pre and post-monsoons, Karayar reservoir, South India

## Abstract

Spatial dataset representing the nutrient distribution in Karayar reservoir during pre and post-monsoon season is presented. Random sampling method was used for data collection and the sample location were fixed using a handheld global positioning system (Garmin GPSMAP-76). The nutrients were estimated using the standard techniques as described in the American Public Health Association (APHA) manual. Physical parameters were estimated using a Hanna portable multi water quality probe (HI-9828, USA). The spatial distribution of physical and nutrient content in surface water is carried out using an inverse distance weighted technique.

## Specifications Table

**Table:** 

Subject area	*Environmental Science*
More specific subject area	*Water quality*
Type of data	*Table, figure*
How data was acquired	Hanna portable multi water quality probe (HI-9828, USA)*, UV–vis Spectrophotometer (DEEP VISION 1371), random sampling, GPS (*Garmin GPSMAP-76*)*
Data format	*Raw, analyzed*
Experimental factors	*The water samples were collected in the first month of each season using an acid washed high-density polyethylene bottles of 1 l capacity.*
Experimental features	*Estimate the concentration of physical parameters and nutrients (temperature, pH, EC, TDS, Cl, NO*_*3*_*, PO*_*4*_*, SO*_*4*_*, NH*_*3*_*N, and DO) in the surface water of Karayar reservoir.*
Data source location	*Tirunelveli, India*
Data accessibility	*Data is within this article*

## Value of the data

• It can sever as a baseline data for the available water-soluble nutrients in the surface water of Karayar reservoir.
• Data shown here can be used to understand the dynamics between forest land use and water quality.
• Data are georeferenced and it can be used in water quality modeling.
• Useful to researchers, policy makers, managers, government officials working in water quality and catchment related fields for protecting the environment.

## 1 Data

**Fig. 1:**
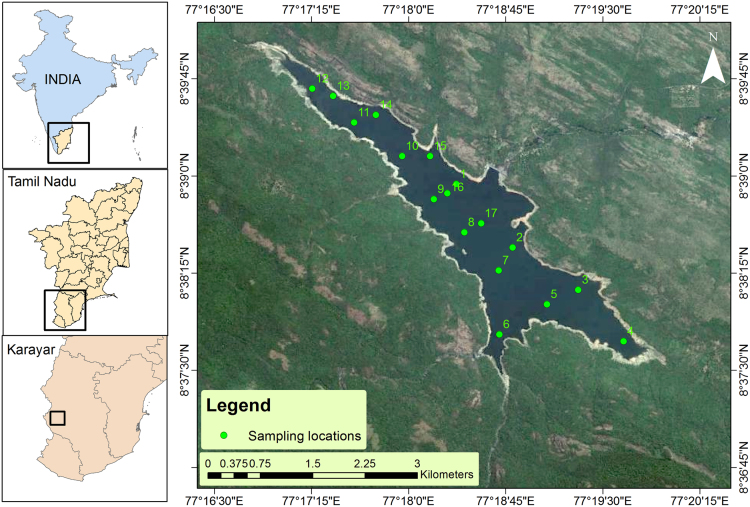
Location map of the study area.

**Fig. 2:**
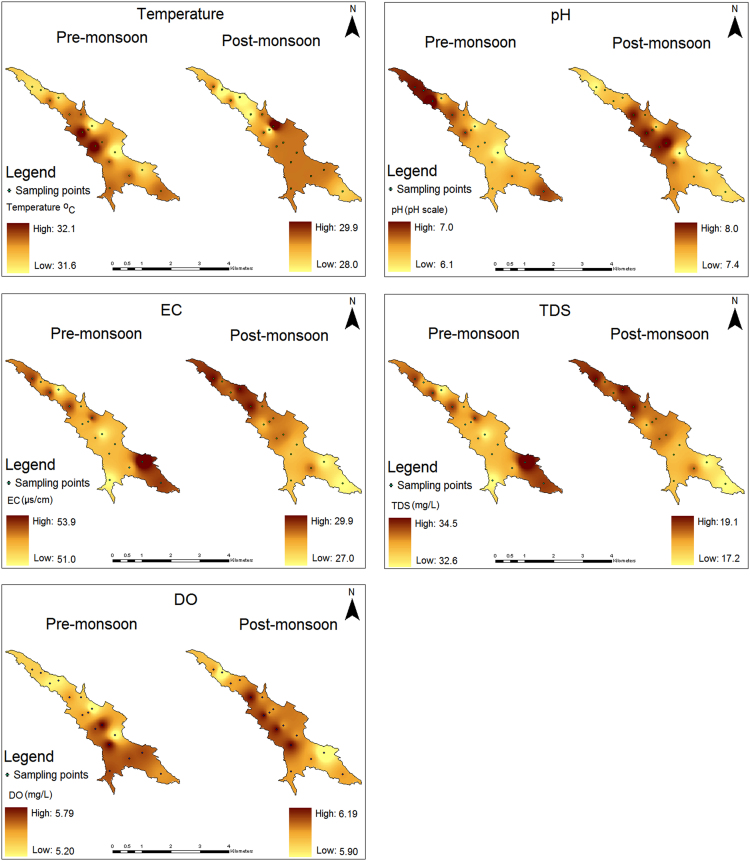
Spatial distribution of physical parameters in the surface water of Karayar reservoir during pre and post-monsoon seasons.

**Fig. 3:**
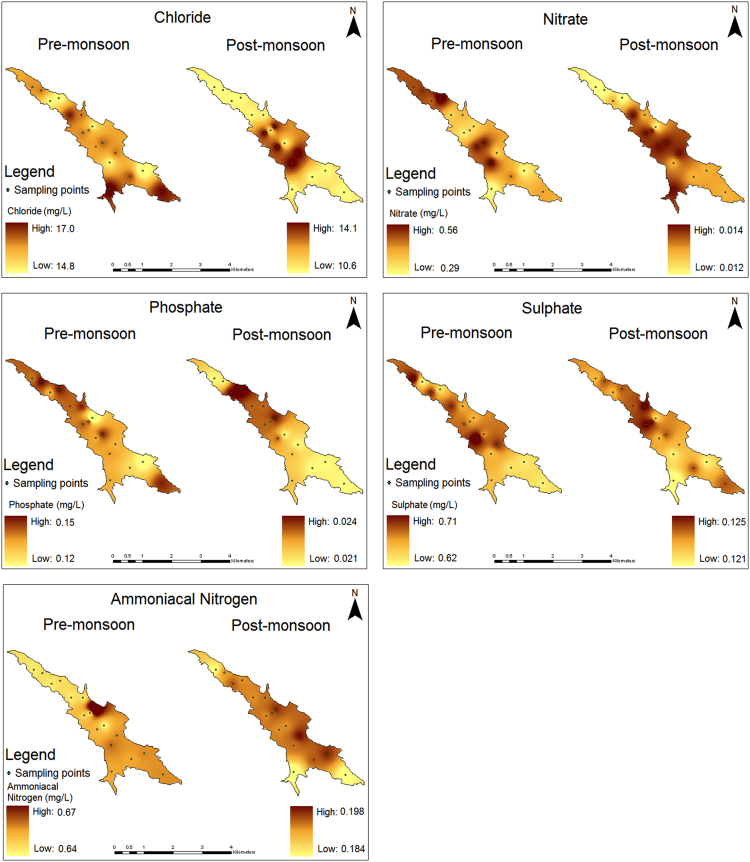
Spatial distribution of nutrients in the surface water of Karayar reservoir during pre and post-monsoon seasons.

The water quality data representing the geographical information, physical and nutrient contents during pre and post-monsoon seasons from 17 locations within Karayar reservoir is shown in Table 1, Table 2. The location map of the study area is shown in Fig. 1. The spatial distribution of all the physical and nutrient contents for both the seasons is shown in Fig. 2, Fig. 3 respectively.

**Table 1:** Physical and nutrient content in the surface water of Karayar reservoir during pre-monsoon season (Temperature in °C, EC in µs/cm, other parameters are shown in ppm).

Sample no.:	Latitude	Longitude	Temperature	pH	EC	Cl^−^	TDS	NO_3_	PO_4_	SO_4_	NH_3_N	DO
1	77.30617	8.648951	31.6	6.2	53	15.2	33.92	0.354	0.12	0.651	0.68	5.2
2	77.31342	8.640801	31.6	6.1	52	16.3	33.28	0.369	0.15	0.682	0.65	5.3
3	77.32184	8.635345	31.8	6.5	54	14.8	34.56	0.321	0.14	0.632	0.65	5.7
4	77.32771	8.628716	32	6.8	53	16.9	33.92	0.421	0.16	0.632	0.65	5.6
5	77.31783	8.633481	32	6.6	52	16.5	33.28	0.439	0.14	0.634	0.65	5.7
6	77.31169	8.629613	32	6.5	51	17.1	32.64	0.296	0.15	0.647	0.65	5.7
7	77.31162	8.637832	32	6.5	52	15.3	33.28	0.531	0.15	0.647	0.65	5.8
8	77.3072	8.642735	32.2	6.5	52	16.1	33.28	0.516	0.15	0.712	0.65	5.7
9	77.30326	8.647017	32.2	6.8	52	16.3	33.28	0.324	0.15	0.671	0.65	5.6
10	77.29919	8.652541	32	6.8	53	16.8	33.92	0.365	0.15	0.681	0.65	5.6
11	77.29298	8.656892	32	7.1	53	15.3	33.92	0.456	0.15	0.682	0.65	5.4
12	77.28759	8.661243	31.8	6.9	53	16.2	33.92	0.471	0.15	0.694	0.65	5.5
13	77.29028	8.660276	31.8	6.9	52	16.3	33.28	0.491	0.16	0.625	0.65	5.5
14	77.29581	8.657859	31.8	6.4	51	15.2	32.64	0.568	0.16	0.632	0.65	5.4
15	77.30278	8.652541	32	6.6	52	16.3	33.28	0.395	0.16	0.654	0.65	5.5
16	77.30499	8.647776	32	6.6	52	16.2	33.28	0.359	0.15	0.658	0.65	5.6
17	77.30934	8.643909	32	6.5	51	16.4	32.64	0.514	0.16	0.659	0.65	5.8
												
		Minimum	31.6	6.1	51	14.8	32.64	0.296	0.12	0.625	0.65	5.2
		Maximum	32.2	7.1	54	17.1	34.56	0.568	0.16	0.712	0.68	5.8
		Mean	31.93	6.61	52	16.1	33.43	0.423	0.15	0.658	0.65	5.56
		Standard deviation	0.17	0.26	0.8	0.67	0.532	0.083	0.01	0.025	0.01	0.17

**Table 2:** Physical and nutrient content in the surface water of Karayar reservoir during post-monsoon season (Temperature in °C, EC in µs/cm, other parameters are shown in ppm).

Sample no.	Latitude	Longitude	Temperature	pH	EC	Cl^−^	TDS	NO_3_	PO_4_	SO_4_	NH_3_N	DO
1	77.30617	8.648951	30	7.6	29	14.2	18.6	0.012	0.024	0.122	0.198	6.1
2	77.31342	8.640801	29	7.4	28	14.2	17.9	0.014	0.021	0.123	0.199	6.1
3	77.32184	8.635345	29	7.6	27	10.7	17.3	0.013	0.021	0.122	0.198	5.9
4	77.32771	8.628716	28.6	7.6	27	10.7	17.3	0.013	0.021	0.124	0.186	5.9
5	77.31783	8.633481	29	7.7	29	10.7	18.6	0.013	0.021	0.124	0.196	6.1
6	77.31169	8.629613	29	7.8	28	10.7	17.9	0.014	0.022	0.121	0.184	6.1
7	77.31162	8.637832	29	7.9	28	14.2	17.9	0.013	0.022	0.122	0.195	6.2
8	77.3072	8.642735	29	8	29	14.2	18.6	0.014	0.023	0.123	0.194	6.2
9	77.30326	8.647017	29	8	28	14.2	17.9	0.014	0.023	0.125	0.194	6.2
10	77.29919	8.652541	28.3	8	30	10.7	19.2	0.014	0.023	0.124	0.194	6.2
11	77.29298	8.656892	29	7.7	29	10.7	18.6	0.013	0.024	0.123	0.196	6.1
12	77.28759	8.661243	29	7.5	30	10.7	19.2	0.012	0.021	0.123	0.187	5.8
13	77.29028	8.660276	28	7.7	29	10.7	18.6	0.012	0.021	0.124	0.194	5.9
14	77.29581	8.657859	28	7.6	30	10.7	19.2	0.012	0.025	0.124	0.194	6.1
15	77.30278	8.652541	29	7.8	29	10.7	18.6	0.013	0.023	0.125	0.195	6.1
16	77.30499	8.647776	28	7.9	29	10.7	18.6	0.014	0.023	0.125	0.196	6.1
17	77.30934	8.643909	29	8.1	29	10.7	18.6	0.014	0.021	0.124	0.194	6.1
												
		Minimum	28	7.4	27	10.7	17.3	0.012	0.021	0.121	0.184	5.8
		Maximum	30	8.1	30	14.2	19.2	0.014	0.025	0.125	0.199	6.2
		Mean	28.82	7.7	28.7	11.7	18.4	0.013	0.022	0.123	0.194	6.071
		Standard deviation	0.51	0.2	0.92	1.67	0.59	8E−04	0.001	0.001	0.004	0.121

## 2 Experimental design, materials and methods

### 2.1 Sample collections

The present study focused at specific water quality monitoring parameters in 17 sampling points in Karayar reservoir. The accurate geographic positions of the sampling points have been determined using a portable global position system—GPS (Garmin GPSMAP-76). The standard methods for analysis of water quality were done as per the guidelines of American Public Health Association [1]. The pre-monsoon water samples were collected in July 2009 and post-monsoon water samples were collected in the month of January 2010. During sampling procedure, the water was sufficiently mixed and remarkable turbulence did not appear. The sampling depth was approximately 30 cm [2]. The water samples were collected in 1-l high-density polyethylene (HDPE) bottles, stored at 4 °C, and further analysed for various chemical parameters in the laboratory. These parameters include temperature, pH, Electrical Conductivity (EC), Total Dissolved Solids (TDS), Nitrate (NO_3_) Phosphate (PO_4_), Sulphate (SO_4_), Ammoniacal Nitrogen (NH_3_-N), Chloride (Cl^-^) and Dissolved Oxygen (DO). The physical parameters such as temperature, pH, EC, TDS, and DO was measured on the spot with the help of Hanna portable multi parameter probe (HI-9828, USA). Chloride was determined by argentometric titration method [1]. NO_3_, PO_4_, NH_3_–N, and SO_4_ were determined by using a UV–vis spectrophotometer at a specified wavelength [1]. All the studied parameters are expressed in ppm except temperature and EC, which is shown in degree celsius and µs/cm respectively.

### 2.2 Geospatial analysis using inverse distance weighted technique

Application of Inverse Distance Weighted (IDW) geographic distribution simulation algorithm was used to build a spatial model using ArcGIS 9.3. According to this algorithm, linear interpolation can be used to interpolate data from sampling points in a restricted neighborhood search area. The attribute of this method is that nearby locations are more likely to have similar values and the linear interpolator weights the interpolated data at unsampled location. The measured parameters shows distinct spatial pattern except the electrical conductivity (EC) and total dissolved solids (TDS). These two parameters shows similar distribution pattern as they are interconnected to each other. The spatial pattern of pH indicates that during premonsoon there is a slight acidic condition exists in the study area, whereas slight alkaline condition persists during the postmonsoon. The dissolution of alkaline earth materials along with rainfall runoff may cause such change in the pH condition. Higher DO concentration in the south-west part of the study area is observed during premonsoon where a major stream inlet (Banatheertham) exists. The water from this stream enhances the DO concentration by naturally aerating the incoming water. During postmonsoon, the spatial pattern (high DO) slightly changes towards the western part of the study area, which is due to the contribution of minor streams from the catchments (Fig. 2). The spatial pattern of phosphate and sulphate shows similar pattern during both the seasons as they may be contributed from source rock materials. Concentration of chloride during premonsoon is observed in the stream inlet points, whereas the pattern changes during postmonsoon. This can be attributed by the dissolution of source rocks during premonsoon and the effect of dilution by rainfall during postmonsoon season. The distribution of nitrate and ammoniacal nitrogen is regulated by mixed origin as these nutrients are derived from biomass decomposition from forests (Fig. 3). The spatial patterns can be used for comparison studies in the future by choosing similar monitoring programs. The point source pollutants through stream inlets can be monitored and its dispersion can be modeled.
