# Supplementary material for: Geospatial analysis of dissolved nutrients dataset in the surface water of Karayar reservoir, Southern India
Source: Data Brief. 2017 Jun 24;13:575–81. doi: 10.1016/j.dib.2017.06.037 (PMC5496461; doi:10.1016/j.dib.2017.06.037)
Supplement: Supplementary file 1 — Supplementary material [file mmc1.docx]

From

**N.S. Magesh**

Researcher

Centre for Geotechnology

Manonmaniam Sundaranar University, Tirunelveli, India

E-Mail: [mageshissivan@gmail.com](mailto:ravisankarphysics@gmail.com)

To

**The Editor**

Data in Brief

Respected Sir

Sub: **Conﬂict of interest – reg.**

The paper entitled “Geospatial analysis of dissolved nutrients dataset in the surface water of Karayar Reservoir, Southern India**”** is submitted for review process and possible publication. I strongly declare that no conﬂict of interest associated with this manuscript.

Thanking You

**Yours faithfully**

**(N.S. Magesh)**
